# Prevalence of posttraumatic arthritis and the association with outcome measures following distal radius fractures in non-osteoporotic patients: a systematic review

**DOI:** 10.1007/s00402-017-2765-0

**Published:** 2017-08-02

**Authors:** C. M. Lameijer, H. J. ten Duis, I. van Dusseldorp, P. U. Dijkstra, C. K. van der Sluis

**Affiliations:** 1 Department of Trauma Surgery, University Medical Center Groningen, University of Groningen, 30.001, Huispostcode BA51, 9700 RB Groningen, The Netherlands; 2 Medical Center Leeuwarden, MCL Academy, Leeuwarden, The Netherlands; 3 Department of Rehabilitation Medicine, University Medical Center Groningen, University of Groningen, Groningen, The Netherlands; 4 Department of Oral and Maxillofacial Surgery, University Medical Center Groningen, University of Groningen, Groningen, The Netherlands

**Keywords:** Wrist, Distal radius, Posttraumatic arthritis, Outcome measures

## Abstract

**Introduction:**

The objective of this systematic review was to analyze (1) prevalence of radiological posttraumatic arthritis (PA), (2) associations of PA with outcome measures and (3) predictors of PA following distal radius fractures in non-osteoporotic patients.

**Materials and methods:**

Nineteen studies were included (10 open source data).

**Results:**

In total, 733 patients were described with a weighted mean age of 37 years (range 25–54) at the time of the injury. Follow-up ranged from 13 months to 38 years. Overall prevalence of PA was 50% and 37% in the open source data. Radial deviation was significantly worse in patients with PA (*N* = 49, mean 14°, SD 6° versus *N* = 55, mean 17°, SD 6°, *p* = 0.037). No analysis could be performed regarding patient reported outcome measures, because of limited data. Articular incongruence was a significant predictor for PA.

**Conclusions:**

A high prevalence of PA was found in non-osteoporotic patients following a distal radius fracture. PA following a distal radial fracture was associated with a limited radial deviation and flexion, but not with grip strength. Articular incongruence predicted PA. Patient reported outcome measures should be investigated more thoroughly to be able to understand the value of using these instruments in interpreting outcome in follow-up of non-osteoporotic patients following a distal radius fracture.

**Level of evidence:**

Level of evidence 3 (Phillips et al. Levels of Evidence—Oxford Centre for Evidence-based Medicine, 1)

## Introduction

Distal radius fractures (DRFs) are common injuries. An annual incidence of 9/10,000 men and 37/10,000 women has been reported in patients aged 35 years and older [2, 3]. In a sample of more than 87 million Americans with an upper extremity fracture in 2009, the most common fracture sites were the distal radius and ulna [4]. The incidence of DRFs is bimodal, with peak incidences in young (predominantly male) patients and older (predominantly females) patients. [4, 5] In young adults this type of fracture results from a high-energy trauma. In older adults, the fracture more often results from low-energy trauma [5, 6]. It has been estimated that at 50 years of age, a woman has 16.6% remaining lifetime risk of sustaining a DRF, whereas a man has a remaining lifetime risk of just 2.9% [7]. Prevalence of radiological posttraumatic arthritis (PA) following DRFs has been described to be as high as 65% after 6.7 years of follow-up [8]. A recent systematic review describing the development of PA suggests that presence of articular steps at the time of healing results in a higher prevalence of radiographic signs of PA [9]. However, the association between radiological PA and clinical outcome remains unclear. Many studies have shown that fractures healed with a step >2 mm are associated with early PA [10–13]. The association between articular incongruence and PA dictates common held beliefs in the treatment of DRFs, where anatomical reduction of the articular surface and stable internal fixation are pursued.

Recent literature supports the hypothesis that increasing age is also an important risk factor for the development of PA [9]. PA is thought to develop less in younger patients. However, it might be of greater importance for this younger age group, because these patients have a long active working and sporting life ahead of them.

### Clinician reported outcome

Function following a DRF can be captured using clinician reported outcomes (CROs), such as range of motion (ROM) and grip strength. PA following a DRF was associated with poorer CROs in some studies [11, 14, 15]. However, other studies did not find this association [16, 17].

### Patient reported outcome

The patient’s opinion regarding wrist function, pain or satisfaction following a DRF can be captured in Patient Reported Outcomes (PROs). A number of studies did not find a significant association between PA and PROs [10, 18, 19]. In contrast, recent literature reported a significant association between presence of PA following DRF and poorer outcomes reported on the SF-36 questionnaire in a heterogeneous age group [20, 21]. Two studies described that a higher age predicted worse PROs 1–6 years following a DRF [20, 22]. The physical component scale of the SF-36 was poorer in older patients. The mental component scale of the SF-36 was similar or even better in younger patients [20, 21, 23]. It has been suggested that patients with preexisting osteoporosis following a DRF have better scores on PROs than those without osteoporosis [21, 24]. Age and/or preexisting osteoporosis seem to be independent factors influencing PROs following a DRF.

### Purpose of the study

Conflicting results in literature have been presented on the association between outcomes and PA following DRFs. There is a need for better understanding of the clinical relevance of radiological PA following a DRF in non-osteoporotic patients. Determination of specific outcome measures predicting PA could be helpful in guiding individual treatment strategies and to decide what rehabilitation goals should be pursued in the follow-up of these patients. In addition, such outcomes could be used to prepare patients on the expected return of function and possible necessary adjustments in everyday life.

The objectives of this systematic review were to analyze (1) the prevalence of PA following a DRF, (2) associations of PA with CROs and PROs and (3) predictors of PA following a DRF in non-osteoporotic patients.

## Methods

### Literature search

A systematic search of the literature was performed in PubMed, Embase, the Cochrane Library and PsycINFO without time restrictions, published until January 2015. The databases were searched with a combination of MesH terms regarding PA, CROs and PROs (Table 1).

**Table 1:** Search strategy in PubMed

Radius fractures	(“Radius Fractures”[Mesh] OR Radius Fracture*[tiab] OR (“Radius”[Mesh] AND “Fractures, Bone”[Mesh]))
Post-traumatic or osteoarthritis	(post traumatic*[tiab] OR posttraumatic*[tiab] OR arthros*[tiab] OR arthrit*[tiab] OR “Joint Diseases”[Mesh] OR osteoarthrit*[tiab])
Functional outcome or patient reported outcome or health status	(“Questionnaires”[Mesh] OR Questionnaire*[tiab] OR short form 36[tiab] OR dash[tiab] OR sf 36[tiab] OR (arm[tiab] AND shoulder[tiab] AND hand[tiab]) OR prwe[tiab] OR patient rated wrist evaluation[tiab] OR mhq[tiab] OR (michigan[tiab] AND hand[tiab] AND outcome*[tiab]) OR “Patient Satisfaction”[Mesh] OR “Patient Satisfaction”[tiab] OR “Pain”[Mesh] OR “Pain”[tiab] OR “Disability Evaluation”[Mesh] OR disabilit*[tiab] OR “Quality of Life”[Mesh] OR qol[tiab] OR “Quality of Life”[tiab] OR life qualit*[tiab] OR range of motion[tiab] OR “Range of Motion, Articular”[Mesh] OR “Recovery of function”[Mesh] OR convalescen*[tiab] OR grip strength[tiab] OR health status[tiab] OR “health status”[Mesh] OR “Health Status Indicators”[Mesh])

Reproduction of the search strategy can be achieved through combining the different sets with the boolean operator AND. The search terms in Embase, the Cochrane Library and PsychInfo were derived from the search terms used in PubMed and are available on request from the author

Eligible for this review were studies reporting adult patients, women between 18 and 49 years, and men between 18 and 59 years at the time of sustaining a DRF. These age selection criteria were applied to eliminate the risk of preexistent osteoporosis, because of the high prevalence of osteoporosis reported in literature in older patients, especially in postmenopausal women [25, 26]. Furthermore, at least one of the CROs (ROM, grip strength) or PROs (Disability of Arm, Shoulder and Hand questionnaire (DASH), Patient Rated Wrist Evaluation (PRWE), Michigan Hand Questionnaire (MHQ), Short Form-36 (SF-36)) had to be described. Follow-up duration had to be at least one year after the DRF. All study designs were included. Excluded were studies with less than 5 patients and studies reporting outcome measures that integrate CROs and PROs, such as the Gartland and Werley score or the Green and O’Brien score, since separate relations of CROs or PROs with PA cannot be established from such measures [21, 27]. Studies describing open fractures were also excluded. Last, studies written in languages other than English, German or Dutch were excluded as were articles that only comprised of a supplement or abstract for a congress.

### Quality assessment

The methodological quality of the selected studies was assessed using the Newcastle–Ottawa Scale (NOS), which is initiated to evaluate the quality of non-randomized studies, such as case–control and cohort studies (Table 2) [28]. The content validity of the NOS has been established based on a critical review of the items by several experts in the field who evaluated its efficiency for assessing the quality of studies to be used in a meta-analysis [28]. With a maximum score of 9, studies were assigned 1 point for each criterion in the checklist that was met, with the exception that 2 points can be assigned in the comparability scale (Table 2) [28]. Studies scoring 75% or more of the maximum score (i.e. >6 points) were considered to be of ‘high quality’. Studies scoring between 50 and 75% (i.e. 5 or 6 points) were labeled ‘moderate quality’. ‘Low quality’ was given to studies with scores lower than 50% (i.e. <5 points). Two reviewers scored the quality (TD, CL), difference in scoring between the two reviewers was resolved with discussion and in case of persistent disagreement a third reviewer (CS) was consulted to reach consensus.

**Table 2:** Newcastle–Ottawa Scale

Category	Item #	Newcastle–Ottawa Quality Assesment Scale—Cohort Studies	Points
Selection	1	Representativeness of the exposed cohort	
		a. Truly representative of the average non-osteoporotic patient with a distal radius fracture in the community	1
		b. Somewhat representative of the average non-osteoporotic patient with a distal radius fracture in the community	1
		c. Selected group of users, e.g. nurses or volunteers	0
		d. No description of the derivation of the cohort	0
	2	Selection of the non-exposed cohort	
		a. Drawn from the same community as the exposed cohort	1
		b. Drawn from a different source	0
		c. No description of the derivation of the non-exposed cohort	0
	3	Ascertainment of exposure	
		a. Secure record (e.g. surgical records)	1
		b. Structured interview	1
		c. Written self-report	0
		d. No description	0
	4	Demonstration that outcome of interest was not present at start of study	
		a. Yes	1
		b. No	0
Comparability	5	Comparability of cohorts on the basis of the design or analysis	
		a. Study controls for posttraumatic arthritis following a distal radius fracture	1
		b. Study controls for any additional factor (this criteria could be modified to indicate control for a second important factor)	1
Outcome	6	Assessment of outcome	
		a. Independent blind assessment	1
		b. Record linkage	1
		c. Self-report	0
		d. No description	0
	7	Was follow-up long enough for outcomes to occur	
		a. Yes (adequate follow-up for posttraumatic arthritis to occur: at least 2 years)	1
		b. No	0
	8	Adequacy of follow-up of cohorts	
		a. Complete follow-up—all subjects for at least 12 months	1
		b. Subjects lost to follow-up unlikely to introduce bias—small number lost <10%	1
		c. Follow-up rate >10% and no description of those lost	0
		d. No statement	1
Total			9 points

A study can be awarded a maximum of one point for each numbered item within the selection and outcome categories. A maximum of 2 points can be given for comparability

### Posttraumatic arthritis assessment

In all studies the classification for PA according to Knirk and Jupiter was applied; grade 0 represents no signs of PA and grade 3 representing bone-on-bone PA with osteophyte and cyst formation [8]. To exclude any chance of bias regarding the severity of PA as graded in the different studies, PA was computed as a dichotomous value; presence or no presence of PA.

### Clinician reported outcome

Range of motion was expressed in degrees. To minimize the effect of the different units of measurement of grip strength (kilograms, kilopascal or pounds), grip strength of the injured wrist as a percentage of the uninjured wrist was calculated. No correction for dominance of hand was performed, in concordance with other studies [14, 29, 30].

### Patient reported outcome

Characteristics of the PROs are described in Appendix 1.

### Data analysis

Regarding reporting data from all studies, associations will be presented when reported in the studies. If associations were presented using Spearman’s *r,* interpretation of effect size was performed using Cohen’s guideline (weak ±0.2, moderate ±0.5, strong ±0.8) [31]. Pooling of open source data was applied to analyze outcomes and associations. The Chi-Square test was used to analyze associations between dichotomous and/or categorical variables. *T* test was used to analyze a dichotomous grouping variable and continuous outcome variables, normal distribution and equality in variances being present. P-plots were used to evaluate normal distribution of data and Levene’s test was used to assess the equality of variances. If there was no normal distribution of data and/or no equality in variances, Mann–Whitney *U* analysis was performed and medians and interquartile ranges (IQR) were presented. In the statistical analysis of the open source data, PA was transformed to a dichotomous variable (presence or no presence of PA). Significance was achieved when *p* < 0.05. All statistical analyses were performed using IBM SPSS, version 22.

## Results

### Study selection

**Fig. 1:**
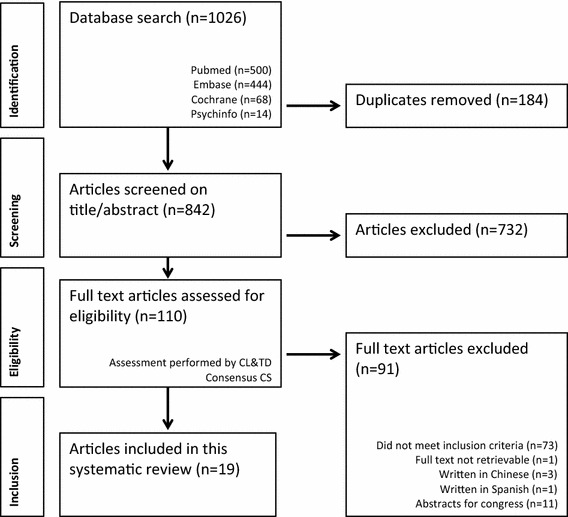
Flowchart of the study selection

The study selection was performed in three stages. First, one reviewer (CL) retrieved 1026 articles from the patient database with the help of an information specialist. All studies where imported in RefWorks^®^. After removing duplicates, a total of 842 studies remained. Second, two reviewers (CL and TD) assessed independently titles and abstracts. A total of 110 papers remained. The same reviewers assessed the full text papers. In case of persistent disagreement a third reviewer (CS) was consulted to reach consensus. Reasons for exclusion were; not retrievable (*n* = 1), written in Chinese language (*n* = 3), written in Spanish (*n* = 1), supplements or abstracts for a congress (*n* = 11) and not meeting the inclusion criteria (*n* = 73). Twenty studies met the inclusion criteria, of which two of the selected publications were conducted by the same research group and described the same patient population [14, 32]. Therefore one of these studies was excluded, resulting in 19 included studies (Fig. 1) [32]. If this was presented in the studies, open source data was collected.

### Study and patient characteristics

#### All studies

The study populations of the included studies ranged from 13 to 106 patients. Eight prospective and 11 retrospective cohort studies were included. A total of 733 patients were described with a weighted mean age of 37 years (range 25–54) at time of the injury. Follow-up ranged from 13 months to 38 years (Table 3). The weighted prevalence of PA was 50% (343 of 683 patients). Seven studies were classified as high quality, nine as moderate quality and 3 of low quality according to the NOS quality assessment (Table 3) [28].

**Table 3:** Characteristics of the included studies

Non open source											
References	NOS	Study type	Eligble *N*/*N*	Mean age (years)	Follow-up (months)	PA	ROM	Grip strength	PRO; mean	Associations with PA	Predictors for PA
Bolmers et al. [18]	5 MQ	PS	47	39	240	Not described	+	+	DASH; B#; 14 C#; 15	ROM: NS Grip strength: NS PRO: NS	–
Foldhazy et al. [36]	7 HQ	PS	37/66	45	108–156	gr I: 8/66	+	+		ROM: NS Grip strength: NS	–
Forward et al. [11]	5 MQ	RS	106	25	456	gr 0: 50 gr I: 31 gr II: 21 gr III: 4	− (% uninjured wrist)	+	DASH; EA# 9 IA# 13	Grip strength: no conclusion VAS: NS DASH: no conclusion	Radial length (OR 1.21, 95% CI 1.02–1.45) Dorsal angulation (OR 1.07, 95% CI 1.03–1.1) Radial inclination: NS Intra-articular fracture (OR 3.23, 95% CI 1.43–7.14)
Goldfarb et al. [34]	7 HQ	RS	16	32	180	gr 0: 0 gr I: 6 gr II: 7 gr III: 2	+	+		ROM: poorer F (*p* < 0.02) Grip strength: NS PRO: NS	
Kopylov et al. [13]	4 LQ	RS	76	31	384 (32 yrs)	33% PA RC 25% PA DRUJ	+	+		ROM: no conclusion Grip strength: no conclusion PA RC ‘more complaints’ than PA DRUJ, no correlation reported	Ulnar variance (2.7 mm with PA vs. 0.9 mm without PA, *p* < 0.01) Dorsal angulation: NS Radial inclination: NS Gender: NS
Lindau et al. [14]	5 MQ	PS	76	41	26	gr 1: 9	+	+		No conclusions	Dorsal angulation: NS Radial shortening: NS Radial inclination: NS Ulnar variance: NS Arthroscopically verified subchondral hematoma
Lutz et al. [15]	7 HQ	RS	81	38	108	gr 0: 2 gr I: 45 gr II: 34	+	+	DASH; 7.5	ROM: F/E: sign lower in gr II vs. gr I PA, (*p* = 0.03) Grip strength: NS VAS: NS DASH: NS	Articular (6/7 patients with stepn >1 mm developed severe PA) Articular cavity depth (4.1 mm gr I PA vs. 5.8 mm gr II PA, *p* < 0.05) AP distance (20.3 mm gr I PA vs.21.7 mm gr II PA, *p* < 0.05) Palmar tilt: NS Radial shortening: NS Radial inclination: NS
Rogachefsky et al. [38]	6 MQ	RS	17	43	30	gr 0: 3 gr I: 10 gr II: 3 gr III: 1	+	+		No conclusions	–
Sharma et al. [17]	8 HQ	PS	64	OP 48 NO 52	24	NO gr I: 16 OP gr I: 5	+	+	DASH; NO 14 OP 5	ROM: NS Grip strength: NS PRO: no conclusion	Less PA in surgically treated patients NO 16 OP 5

Open source											
References	NOS	Study type	Eligble *N*/*N*	Mean age (years)	Follow-up (months)	PA	ROM	Grip strength	PRO; mean	Associations with PA	Predictors for PA
Beyermann [37]	6 MQ	RS, POS (no PA)	10/19	53	32.5	All <gr II	+	+	DASH; 11.5	No conclusions	–
Catalano et al. [10]	5 MQ	RS, POS	21	30	86	gr 0: 5 gr I: 10 gr II: 6	+	+		ROM: supination (rho = −0.476, *p* = 0.029) Grip strength (*r* = 0.463, *p* = 0.034) PRO: NS	Step (*r* = 0.74, *p* < 0.001) Gap (*r* = 0.70, *p* < 0.001)
Doi et al. [33]	7 HQ	PS, OS	33/82	52	31	gr 0: 16 gr I: 10 gr II: 6 gr III: 1	+	+		ROM: F (*r* = −0.350, *p* = 0.046) Grip strength: no conclusion	Incongruency (*p* < 0.001) Arthroscopically assisted reduction less PA (*p* = 0.014)
Espen [19]	5 MQ	RS, OS	11/20	53	38	gr 0:3 >gr 0:8	+	+	DASH; 20.5	ROM: NS Grip strength: NS PRO: NS	–
Fernandez et al. [12]	8 HQ	RS, OS	31/50	49.6	28.8	1/31	–	–	SF-36; PC: 46.58 MC: 53.06	PRO: With PA significant lower PC score than without PA (27.9 vs. 48.2, *p* < 0.001)	Incongruency (*p* < 0.005)
Fitoussi et al. [35]	4 LQ	PS, OS	31/34	42	24	gr 0: 23 gr I: 2 gr II: 4 gr III: 2	+	+		ROM: F (*r* = −0.429, *p* = 0.016) Supination (*r* = −0.423, *p* = 0.018) Grip strength: no conclusion	Incongruency >2 mm (*p* < 0.05)
Jupiter and Lipton [29]	5 MQ	RS, OS	10/13	35	30	gr 0: 9 >gr II: 1	+	+		No conclusions	–
Ring et al. [16]	7 HQ	PS, OS	18/25	46	26	gr 0: 13 gr I: 4 gr II: 1	+	+		ROM: NS Grip strength: NS	–
Strange-Vognsen [39]	4 LQ	RS, POS	42	29	187	gr 0: 15 gr I: 11 gr II: 9	–	± (only reduced y/n)		No conclusions	Residual deformity (*r* = 0.34, *p* < 0.05) Step (*r* = 0.34, *p* < 0.05) Dorsal angulation: NS Radial inclination: NS
Tyllianakis et al. [30]	5 MQ	PS, POS	6/21	53.5	13	gr 0: 3 gr I: 2 gr II: 1	+	+		No conclusions	–
Total	7 HQ 9 MQ 4 LQ	7 OS 3 partially OS	733 (OS 213)	37 yrs (range 25–54)	range (13-456)	gr 0: 142 > gr 0: 343	15	17	9		

*PA* posttraumatic arthritis (grading according to Knirk and Jupiter [8]), *ROM* range of motion, *PRO* patient reported outcome measure, *DASH* disability of arm, shoulder and hand questionnaire, *NOS* Newcastle-Ottawa Scale, *LQ* low quality, *MQ* medium quality, *HQ* high quality, *PS* prospective, *RS* retrospective, *MA* meta-analysis, *OS* open source, *POS* partially open source, *EA* extra-articular, *IA* intra-articular, *NO* non-operative, *OP* operative, *NS* not significant, *F* flexion, *E* extension, + the study describes the outcome measurement described, − the study does not describe the outcome measurement described, *OR* Odds ratio, 95% *CI* 95% confidence interval

#### Open source

Ten studies comprised of open source data of 213 patients (169 men) with a median age of 37 years (IQR 27; 44) and median follow-up of 31 months (IQR 24; 73) (Table 4). The classification of the fracture type was described in 161 patients, with the majority having an AO/OTA type C3 fracture (*n* = 74). The weighted overall prevalence of PA was 37%. Prevalence of PA after a follow-up of ≤36 months (range 12.5–36 months) was 31%. This was statistically significantly lower than the prevalence of PA (64%, *p* < 0.001) after a follow-up of >36 months (range 36–192 months).

**Table 4:** Patient characteristics from the open source data

Characteristics	PA (*N* = 79)	No PA (*N* = 117)	Total population (*N* = 213)		Significance
	N/median (IQR)	N/median (IQR)	N/median (IQR)	%	
Age (years)					
*N*	79	117	213		NS
Median	36 years (27–44)	37 years (27–44)	37 yrs (27–44)		
Gender					
Male	64	92	169	79	NS
Female	15	24	44	21	
Follow-up (months)					
*N*			193		*p* = 0.026
Median	46 months (24–100)	28 months (24–37)	31 months (24–73)		
Trauma energy					
Low energy	7	4	15	21	NS
High energy	30	25	58	79	
AO/OTA fracture classification					
A	3	11	11	6.4	NS
B	56	10	13	7.6	
C	59	81	147	86	
Treatment					
Non-operative	18	14	38	18	*p* = 0.047
Surgery	61	102	174	82	
Articular incongruence at follow-up (step and/or gap)					
No	8	58	66	48%	*p* < 0.001
Yes	51	20	71	52%	
Step (mm) median	1.0 mm (1.0–2.0)	0.0 mm (0.0–1.0)	1.0 mm (0.0–1.7)		*p* < 0.001
No	9	25	34	39	
Yes	44	10	54	51	
Gap (mm) median	0.0 mm (0.0–1.0)	0.0 mm (0.0–0.0)	0.0 mm (0.0–1.0)		*p* = 0.017
No	20	19	39		
Yes	13	2	15		
Grading PA according to Knirk and Jupiter					
Gr 0		84	84	55	
Gr I	39		39	25	
Gr II	28		28	18	
Gr III	3		3	2	

*IQR* interquartile range,* PA* posttraumatic arthritis,* NS* not significant,* mm* millimeter

### Association between PA and CRO: range of motion

#### All studies

Three out of the 16 studies describing ROM, reported a statistically significant association between the presence of PA and diminished flexion (Table 3) [33–35]. Two of these three studies described moderate statistical significant associations (*r* = 0.350, *p* = 0.046 and *r* = 0.429, *p* = 0.016, respectively) [33, 35]. One study described a statistically significant lower ROM in flexion–extension arc in patients with PA grade II compared to patients with grade I PA [15]. A moderate statistically significant association between PA and poorer supination was found in one study (*r* = −0.476, *p* = 0.029) [10]. Five studies did not find a statistically significant association between PA and ROM. [16–19, 36] In the six remaining studies the association between PA and ROM was not analysed [14, 29, 30, 37–39].

#### Open source

Of the 10 studies with (partially) open source data, seven had data regarding ROM (Table 3) [10, 16, 19, 29, 33, 35, 37]. Pooled data analysis is presented in Table 5. Radial deviation was statistically significantly worse in the patients with PA (*N* = 49, mean 14°, SD 6°) compared to patients without PA (*N* = 55, mean 17°, SD 6°, *p* = 0.037). All other outcomes regarding range of motion in the patients with and without PA did not differ with statistical significance (Table 5).

**Table 5:** Open source data regarding ROM and grip strength

Range of motion		PA			No PA			Significance
	*N*	Mean (°)	SD	*N*	Mean (°)	SD	*N*	
Flexion	124	52	18	55	52	16	69	NS
Extension	124	53	13	55	54	11	69	NS
Arc motion F/E	124	105	26	55	107	23	69	NS
Ulnar deviation	104	23	9	49	25	8	55	NS
Radial deviation	104	14	6	49	17	6	55	*p* = 0.037
Arc motion UD/RD	104	37	12	49	42	11	55	*p* = 0.063
Pronation	124	76	14	55	75	12	69	NS
Supination	124	76	15	55	81	19	69	NS

Grip strength		PA			No PA			Significance
	*N*	Mean (%)	SD		Mean (%)	SD	*N*	
% Grip strength	124	79	18	55	79	15	69	NS

*SD* standard deviation, *PA* posttraumatic arthritis, *NS* not significant, *F/E* dorsal flexion/extension, *UD/RD* ulnar deviation/radial deviation, ° degrees, % percentage

### Association between PA and CRO: grip strength

#### All studies

One out of the 18 studies describing grip strength found a moderate statistically significant association between severity of PA and diminished grip strength (*r* = 0.464, *p* = 0.034) [10]. In contrast, seven studies did not find a significant association [15–19, 34, 36]. The remaining 10 studies did not analyse the association between PA and grip strength.

#### Open source

Eight studies presented open data regarding grip strength (Table 3) [10, 16, 19, 29, 30, 33, 35, 37]. No statistically significant association between PA and grip strength was found (Table 5).

### Association between PA and PROs

PROs were reported in few studies (Table 3). It was decided not to report nor perform statistical analysis on these limited results.

### Predictors of PA

#### All studies

Eleven studies reported on predictors of PA following a DRF (Table 3). Articular incongruence (step and/or gap) at follow-up was found to be a statistically significant predictor of PA in six studies [10, 12, 15, 33, 35, 39]. The weights of the associations were described as strong and moderate in two of these 11 studies (step *r* = 0.74, *p* < 0.001 and gap *r* = 0.70, *p* < 0.001; step *r* = 0.34, *p* < 0.05, respectively) [10, 39]. Conflicting results on other predictive radiological factors, such as shortened radial length, dorsal angulation, radial inclination, ulnar variance and AP distance, were reported (Table 3) [11, 14, 39, 40]. In a longitudinal study a significant progression of PA after a longer follow-up duration was found (15 vs. 7 years, *p* = 0.02) [34]. Older age at the time of injury was associated with earlier development of PA [11]. Gender was not associated with the development of PA [13]. One study described PA to be statistically significantly less often present in patients treated surgically compared to patients treated conservatively [17]. Another study reported less PA when arthroscopically assisted surgical treatment was performed compared to non-arthroscopically assisted surgical treatment [33].

#### Open source

At a median follow-up of 31 months (IQR 24; 73) 52% of the patients had some kind of articular incongruence (step and/or gap). Patients with PA experienced statistically significant more often residual articular incongruence in comparison to patients without PA (51 versus 20 patients, *p* < 0.001). Furthermore, patients with PA experienced statistically significant more often a residual step (44 versus 10 patients, *p* < 0.001) or gap (13 versus 2 patients, *p* = 0.017) (Table 4). Follow-up was statistically significant longer in the patients who did develop PA (median 46 months (IQR 24; 100) versus median 28 months (IQR 24; 37), *p* = 0.026). All possible radiological predictors directly after fracture reduction and at the end of follow-up were not significantly associated with PA (Table 6).

**Table 6:** Open source data regarding predicting radiological factors for PA

Radiological measurement	*N*	PA			No PA			Significance
		Mean	SD	*N*	Mean	SD	*N*	
Dorsal angulation (°)								
Postreduction	31	1	7.6	8	−3.3	9.4	23	NS
Follow-up	149	−1.3	10.2	58	−2.7	9.3	91	NS
Radial length (mm)								
Postreduction	33	10.1	5.2	8	9.9	3.7	23	NS
Follow-up	81	10.6	3.9	25	10.8	3.6	56	NS
Ulnar variance (mm)								
Postreduction	27	1.4	2.1	7	−0.2	2.0	20	NS
Follow-up	98	1.0	2.3	45	0.9	2.3	53	NS
Radial inclination (°)								
Postreduction	31	21.3	8.9	8	19.7	5.7	23	NS
Follow-up	148	21.5	5.1	57	21.2	4.7	91	NS

*SD* standard deviation, *PA* posttraumatic arthritis, *NS* not significant, ° degrees, % percentage

Age at the time of injury did not differ statistically significantly between patients with and without PA (Table 3). Gender was not associated with the presence of PA. No statistical analysis on the influence of intra- versus extra-articular fracture types on PA could be performed, because only 11 patients with an extra-articular fracture were described. In the patients with intra-articular fractures, no significant difference in the development of PA was seen between AO/OTA type B and C fractures or between AO/OTA type C1, C2 or C3 fractures (Table 4).

## Discussion

A high prevalence of the development of PA following a DRF in non-osteoporotic patients was found (50% in all patients with a range in follow-up duration of 13 months to 38 years, 37% in the open source studies after a median follow-up of 31 months). In addition, this study shows that the prevalence of PA seems to worsen over time (respectively, 31% after a follow-up of 0–36 months versus 64% follow-up duration after 36 months). Presence of PA was statistically significantly associated with diminished radial deviation and flexion, but not with grip strength. Unfortunately, no conclusions could be drawn regarding the association between PA and PROs, because of lack of data. An intra-articular step or gap had a statistical significant negative effect on the development of PA. No further associations between radiological predictors and PA were found using open source data. Operative treatment or arthroscopically assisted surgical treatment seemed to reduce the chance of developing PA [17, 33].

### Prevalence of PA

The high prevalence of PA in this non-osteoporotic population is worrisome. However, from the included studies we could not derive sufficient information on the restrictions or limitations these patients experienced when executing activities of daily living, leisure time activities, work or other societal roles. Further research on PA in non-osteoporotic patients with DRF should elaborate on the impact of PA on patients’ activities or participation. Since most studies comprise of small study populations and because the open source data showed that a longer follow-up duration is associated with a higher prevalence of PA, specifically open source studies may provide unique chances to gather such data. However, currently no uniform set of evaluation instruments is available, which results in difficulty of pooling data.

### Association between PA and CROs

Wrist motion is dependent on complex articulations of the scaphoid, lunate and the radio carpal joint [41]. Biomechanically, flexion–extension and radio-ulnar deviation are a result of motion of the scaphoid and lunate in respect to the distal radius, which relies on the ligamentous stability between these two carpal bones and movement in the adjacent joint surfaces [42]. The majority of the DRFs in non-osteoporotic patients result from high-energy trauma and, therefore frequently are intra-articular fractures. It is imaginable that the direction of the intra-articular force associated with this type of fracture causes intercarpal ligamentous injury as well as joint surface changes. Recent literature describes an incidence of 38% of associated scapholunate (SL) or lunotriquetral (LT) ligamentous injuries in distal radius fractures. [43] Associated SL or TL ligamentous injuries could be an explanation for the limited radial deviation and flexion and early PA as described in this systematic review. Furthermore, malalignment of the distal radius following a fracture can cause alterations of the distal radio-ulnar joint with anatomical change of the radio-ulnar contact area, resulting in limited pronation and supination [44]. Based on the results of this systematic review, it can be concluded that grip strength does not seem to be influenced by PA. This emphasizes that grip strength might not be an important determinant of wrist function and is not one of the first symptoms of PA, but merely a reflection of overall muscle strength and condition [45]. Ageing is typically associated with a progressive loss of skeletal muscle mass and occurs at a rate of 3–8% each decade after the age of 30 years [46, 47]. Although age is a confounding factor for grip strength, our results indicate that in this relatively young group of patients grip strength is not influenced to a significant extent by age. A recent Cochrane reported on different rehabilitation methods following distal radius fractures in adults was published [48]. Twenty-six trials were included which turned out to be inhomogeneous with regards to patient characteristics (i.e. age) and were qualified as low or very low quality evidence. The authors therefore concluded that available evidence is insufficient to establish the relative effectiveness of different rehabilitation methods. It is suggested by the authors to precede rehabilitation research regarding outcome in patients with distal radius fractures with a clear aim [48]. From our systematic review it is suggested that rehabilitation in non-osteoporotic patients with distal radius fractures should have a broad approach, with special focus on wrist motion (radial deviation and flexion). Although we did not find an association between grip strength and radiological PA, it is still an important determinant of total outcome and should be addressed appropriately in rehabilitation treatment.

### Associations between PA and PROs

No conclusions could be drawn regarding the association between PA and PROs, because of limited data. This is indicative of a gap in knowledge on the clinical relevance of radiological PA as measured by PROs, despite the high prevalence of PA in this group.

### Predictive factors for PA

A high prevalence of PA was shown, and a longer follow-up duration was associated with a higher prevalence of PA. As such, development of PA seems to be a dynamic process and progresses over time. In addition, articular incongruence was predictive for PA: patients with a step or a gap had a higher prevalence of PA. This outcome resembles the conclusions drawn in studies regarding associations between articular incongruence and PA following a DRF [10–13]. When articular incongruence is associated with the development of PA, it might be assumed that the AO/OTA classification of the fracture type would also have an association with PA. This association was not found in this study. The reason no statistical significant differences were found between AO/OTA type B and C fractures regarding the development of PA could be that inter- and intraobserver variability of the AO/OTA classification of distal radius fractures has been reported to be moderate to poor [49]. Another explanation could be that the DRFs have been surgically treated if a large incongruence was present and only the residual deformity or articular incongruence will affect the development of PA. It is hypothesized that with surgical treatment (with or without direct arthroscopic control), better anatomical reduction of the articular surface can be achieved and, therefore may diminish the chance of developing PA [17, 33]. Conflicting results regarding several radiological measurements predicting PA were presented in literature. However, analysis of our open source data suggests that dorsal angulation, radial length, ulnar variance and radial inclination do not predict PA. These conflicting results on predicting radiological factors could be due to a substantial variability in how these factors are defined in literature. It has been suggested to develop guidelines to ensure consistency when interpreting different radiographic measurements reported in literature [50].

### Strength and weaknesses

This study is the first systematic review presenting CROs and PROs and the association with PA following DRFs in non-osteoporotic patients. Because of the extent of this systematic review and the pooling of the open source data, we believe this study is a contribution to the insight in the prevalence and clinical relevance of PA in non-osteoporotic patients following a DRF. Recent literature has encouraged pooling of open source data from clinical trials and cohort studies and reporting this in systematic reviews and meta-analyses to compare outcome in a more reliable and efficient manner [51, 52]. Although we believe pooling of the open source data in this systematic review contributes to the strength of the conclusions, variability between raters and the way measurements have been performed, should be acknowledged. Some other limitations of our systematic review should be acknowledged. We have chosen an age selection criteria (men 18–59 years, women 18–49 years) to eliminate the risk of preexisting osteoporosis. Despite our selection criteria, some of the included patients may still have had osteoporosis. All studies included in this systematic review were cohort studies or case–control studies (level of evidence II and III) with relatively small populations and moderate methodological quality [53]. These restrictions should be taken into account when interpreting the results of this meta-analysis. In general, research in the field of rehabilitation and injuries should be more transparent by presenting open source data, especially when describing small populations, to be able to compare data in a reliable way. In addition, despite our extensive literature search, very limited data was retrieved regarding PROs. We decided not to report on these limited results and, therefore no conclusions could be drawn regarding PROs following DRFs in non-osteoporotic patients. Furthermore, a new scoring method was used to assess the methodological quality of the studies, with equal weights for each quality category, except for the comparability category [28]. It could be argued that the quality categories should be scored separately instead of a combined total score to provide optimal insight into the quality of the different studies. Most included studies reported statistical significance of their results, but the weight of the associations was poorly described. Several authors have described associations between the residual articular incongruence and PA.

### Further research

The high prevalence rate of PA found in the (pooled open source) data shows that investigation of outcome in non-osteoporotic patients with a long active and working life ahead should have more attention. To direct treatment strategy, rehabilitation and to decide what an acceptable level of rehabilitation is in the follow-up of non-osteoporotic patients with a DRF there is a need for a reliable interpretation of PROs and the association with PA investigated by using randomized controlled trials with or without implementing pooling of open source data. For patients and therapists it would be of great value to be able to work towards an evidence-based rehabilitation goal. It would also be very beneficial to gain more insight in the influence of radiological characteristics following fracture reduction, such as radial shortening and radial inclination on CROs, PROs and PA.

## Conclusions

Half of all non-osteoporotic patients developed some degree of PA following a DRF. In addition, PA seems to progress over time. PA following a distal radial fracture was associated with a limited radial deviation and flexion, but not with grip strength. This suggests that rehabilitation should have a broad approach, with focus on wrist motion, and on learning to adjust daily activities to limited wrist motion to optimize functional recovery. Unfortunately no conclusions could be drawn regarding PROs and their clinical applicability in the follow-up of DRF in non-osteoporotic patients, because of limited data. PROs should be investigated more thoroughly to be able to understand the value of using these instruments in interpreting outcome in follow-up of these non-osteoporotic patients. Further research could produce evidence-based rehabilitation goals for patients and therapists. Treatment of DRF should be directed at avoiding articular incongruence, because of its statistically significant association with the development of PA. Conflicting results in literature have been reported on dorsal angulation, radial length, ulnar variance and radial inclination on predicting PA. More thorough research on other radiological factors predicting PA could show more insight on primary treatment goals to avoid PA in the follow-up of these young non-osteoporotic patients.

## Appendix 1. Specific characteristics of PROs

DASH. The Disability of Arm Shoulder Hand questionnaire is a 30-item self-report measure focusing on physical functioning and symptoms of the upper limb. DASH scores range from 0 to 100 (higher scores indicate worse function).

PRWE. The Patient Rated Wrist Evaluation assesses pain and functioning in patients with wrist fractures [54]. The PRWE includes 5 items assessing pain, which are rated from 0 (no pain) to 10 (unbearable pain) and 10 items assessing function [36]. The function subscale is divided into two sections concerning specific activities and usual activities. For each section the maximum score is 50 (most disability) and the minimum score is 0 (no disability). A higher score indicates a worse outcome. The questionnaire has a fair validity for symptoms and function of the wrist.

MHQ. The Michigan Hand Outcomes Questionnaire is a validated questionnaire assessing hand outcomes that are of importance to patients and specific for the impaired hand (left and right separately). It includes 6 domains (overall hand function, activities of daily living, pain, work performance, aesthetics and satisfaction). A higher score indicates a better function of the impaired wrist [55].

SF-36. The Short Form-36 questionnaire is developed to survey overall health status [56]. It uses 36 items to asses limitations in (1) physical function, (2) role function, (3) social function, (4) bodily pain, (5) general mental health, (6) limitations in role function due to emotional problems, (7) vitality and (8) general health perception. A physical and a mental component summary score can be calculated. A higher score indicates a better quality of life as experienced by the patient.
