# Correction to: Prevalence of posttraumatic arthritis and the association with outcome measures following distal radius fractures in non‑osteoporotic patients: a systematic review

**DOI:** 10.1007/s00402-021-04117-2

**Published:** 2021-09-14

**Authors:** C. M. Lameijer, H. J. ten Duis, I. van Dusseldorp, P. U. Dijkstra, C. K. van der Sluis

**Affiliations:** 1 Department of Trauma Surgery, University Medical Center Groningen, University of Groningen, 30.001, Huispostcode BA51, 9700 RB Groningen, The Netherlands; 2 Medical Center Leeuwarden, MCL Academy, Leeuwarden, The Netherlands; 3 Department of Rehabilitation Medicine, University Medical Center Groningen, University of Groningen, Groningen, The Netherlands; 4 Department of Oral and Maxillofacial Surgery, University Medical Center Groningen, University of Groningen, Groningen, The Netherlands

## Correction to: Arch Orthop Trauma Surg (2017) 137:1499–1513 10.1007/s00402-017-2765-0

Unfortunately, the given name and family name of the third author was incorrectly tagged in the xml data, therefore it is abbreviated wrongly as “Dusseldorp IV” in PubMed. The correct given name is Ingeborg and family name is van Dusseldorp.

